# Metachronous Double Primary Malignancies of Invasive Ductal Carcinoma of the Breast and Primary Ovarian Angiosarcoma

**DOI:** 10.22034/ijp.2026.2077093.3577

**Published:** 2026-08-01

**Authors:** Noviana Nugrohowati, Lely Amedia Ratri, Tania Kusuma, Irianiwati Widodo

**Affiliations:** 1 Department of Anatomical Pathology, Faculty of Medicine, Public Health, and Nursing, Universitas Gadjah Mada, Yogyakarta, Indonesia; 2 Department of Anatomical Pathology, Academic Hospital Universitas Gadjah Mada, Yogyakarta, Indonesia

**Keywords:** Ovarian neoplasm, angiosarcoma, breast neoplasm

## Abstract

**Background & Objective:**

Primary ovarian angiosarcoma (POA) is an exceptionally rare and aggressive endothelial malignancy, accounting for less than 1% of all ovarian cancers. Its diagnosis is often challenging due to nonspecific clinical features and histologic overlap with other ovarian tumors.

**Case Presentation:**

A 47-year-old woman presented with progressive abdominal distension, intermittent vaginal bleeding, and constipation, four months after completing chemoradiotherapy for invasive ductal carcinoma of the breast. Imaging revealed a 14.6 cm solid-cystic pelvic mass, and histopathological examination demonstrated CD31 and ERG positivity, confirming a rare diagnosis of primary ovarian angiosarcoma. BRCA2 immunohistochemistry was negative.

**Conclusion:**

The metachronous occurrence of triple-negative invasive ductal carcinoma and primary ovarian angiosarcoma represents an extremely rare clinical scenario. Although angiosarcomas have occasionally been reported in individuals with BRCA mutations, the relationship between BRCA alterations and ovarian angiosarcoma remains unclear. In the present case, immunohistochemical analysis demonstrated negative BRCA2 expression. Further molecular characterization of ovarian angiosarcomas is needed to better understand their biology and potential relationship with hereditary cancer syndromes.

## Introduction

Breast cancer is the most frequently diagnosed malignancy among women, accounting for approximately 23% of all female cancers worldwide, and together with ovarian cancer represents a leading cause of cancer-related mortality.(1,2) The occurrence of both breast and ovarian malignancies, whether synchronous or metachronous, in a single patient is uncommon and often suggests an underlying hereditary predisposition, warranting evaluation for genetic or molecular abnormalities. Most reported cases, however, involve epithelial ovarian carcinomas rather than mesenchymal or vascular tumors. Reports describing the coexistence of breast carcinoma and mesenchymal ovarian malignancies are extremely limited.

While most ovarian cancers are epithelial in origin, a small subset arises from non-epithelial elements, including germ cell and mesenchymal tumors. Among these mesenchymal tumors, ovarian angiosarcoma (OA) represents an exceptionally rare and aggressive endothelial malignancy, accounting for less than 1% of all ovarian cancers.(3) Fewer than 60 cases have been reported in the English literature, reflecting the extremely rare occurrence of this condition. Its etiology remains unclear, though prior radiation, exposure to vinyl chloride or arsenic, and chronic lymphedema have been implicated. Diagnosis is challenging due to nonspecific clinical features and histologic overlap with other ovarian malignancies. Due to its rarity, there is no standardized treatment protocol for primary ovarian angiosarcoma. Most patients undergo surgical resection followed by adjuvant chemotherapy, although reported responses are variable.(4)

Germline mutations in the BRCA1 and BRCA2 genes represent the most well-established genetic link between breast and ovarian malignancies and are responsible for hereditary breast and ovarian cancer syndrome (HBOC).(5,6) BRCA1 and BRCA2 are both tumor suppressor genes that, when mutated, significantly increase lifetime risks for breast, ovarian, and other cancers, but they differ in risk levels and cancer types.(6) BRCA1 mutations typically present higher risks for earlier-onset, triple-negative breast cancer and ovarian cancer. BRCA2 mutations are more associated with hormone receptor-positive breast cancer, male breast cancer, and higher risks of prostate or pancreatic cancer. Although HBOC has traditionally been associated with epithelial ovarian carcinomas, the relationship between BRCA mutations and non-epithelial ovarian malignancies remains unclear. Primary ovarian angiosarcoma is an exceptionally rare tumor, and its potential association with hereditary cancer syndromes has not been clearly established. In this context, the coexistence of distinct tumor types in a single patient is notable, although any potential shared biological mechanisms remain unclear in the absence of molecular confirmation.

This case report describes a patient who developed metachronous triple-negative invasive ductal carcinoma of the breast followed by primary ovarian angiosarcoma, an extremely rare clinical scenario. This case contributes to the limited literature on rare dual malignancies and highlights the need for further investigation to better characterize the pathogenesis of ovarian angiosarcoma and to clarify whether any relationship exists between these two malignancies.

## Case Presentation

A 47-year-old G1P0A1 female was referred to our hospital for chemotherapy, having previously undergone a modified radical mastectomy of the left breast at another institution, where histopathological examination confirmed invasive ductal carcinoma, staged T3N0Mx. A family history of malignancy, smoking, hormonal therapy, and contraceptive use was denied. Physical examination, vital signs, and laboratory results at presentation were unremarkable.

Repeat immunohistochemistry (IHC) of the breast tumor demonstrated estrogen receptor (ER)-negative, progesterone receptor (PR)-negative, and HER2-negative expression, with a Ki-67 proliferation index of 30%, consistent with triple-negative breast cancer (TNBC). She subsequently received 4AC-4T chemotherapy administered every three weeks, followed by 25 fractions of radiotherapy. Treatment was generally well tolerated, with only mild nausea, fatigue, and transient dysgeusia, although she experienced a 7-kg weight loss. Post-treatment ultrasound of the breast and chest wall revealed no residual or recurrent tumor and no lymphadenopathy.

**Fig. 1:**
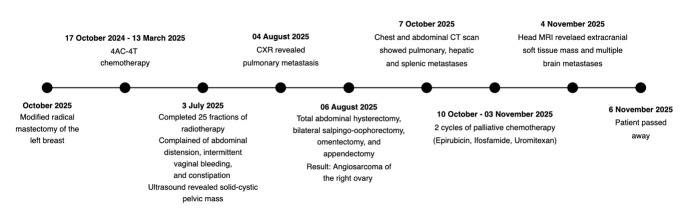
Clinical timeline illustrating the patient’s medical history from the treatment of breast cancer until her death.

Several days after completing radiotherapy, the patient reported progressive abdominal distension, intermittent vaginal bleeding, and constipation that had been ongoing for the past two months. Physical examination revealed a large, firm, non-tender mass extending from the suprapubic region to three fingers above the umbilicus, with restricted mobility. Abdominal and pelvic ultrasound demonstrated a 14.6 × 10.5 × 15.4 cm solid-cystic pelvic mass, likely of gynecologic origin, with associated left renal pelviectasis and a renal cyst. The patient’s past medical history was notable for a miscarriage treated with curettage in 2010 and a left ovarian endometriotic cyst managed by laparotomy in 2017 (Fig. 1).

**Fig. 2:**
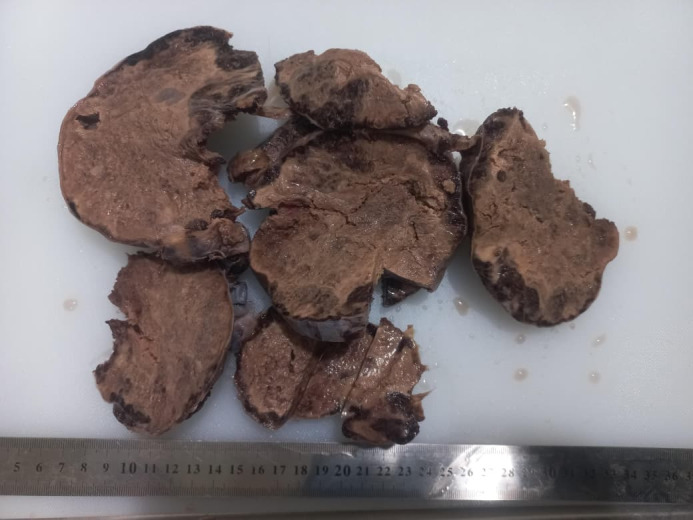
Gross appearance of the ovarian mass, showing a friable, dark brown to black solid tumor with focal areas of necrosis and hemorrhage.

**Fig. 3:**
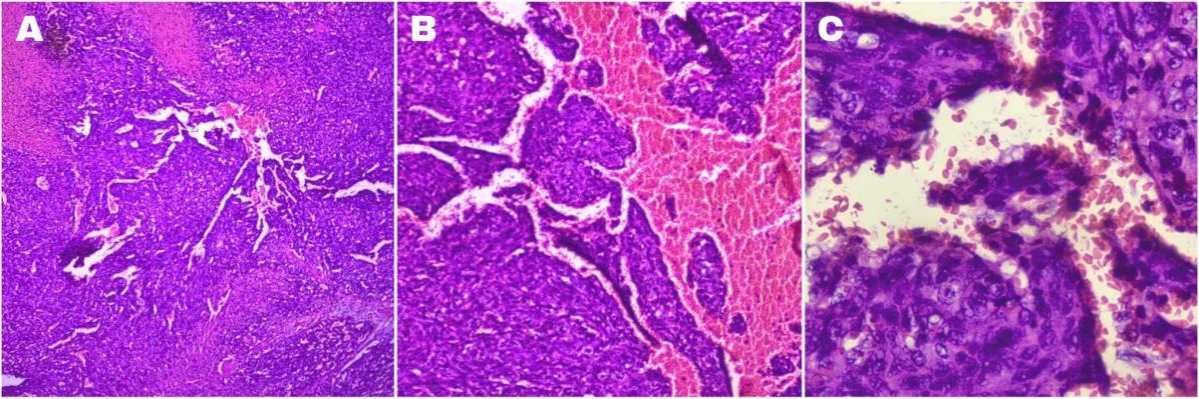
Hematoxylin and eosin (H&E)-stained sections. A. Low-power magnification (40×) showing ovarian parenchyma along with the vascular lesion and papillary vascular channels. B. Medium-power magnification (100×) showing a highly cellular, irregular neoplasm composed of proliferating, interconnected vascular channels, ranging from focal or diffuse vasoformative components with dilated, slit-like, papillary, or interconnecting vascular spaces to solid components that may be spindle sarcomatous or epithelioid. C. High-power magnification (400×) revealing tumour cells exhibiting marked pleomorphism and lining vascular lumina containing erythrocytes. The cells display atypical features with round, oval, or spindle-shaped hyperchromatic nuclei.

**Fig. 4:**
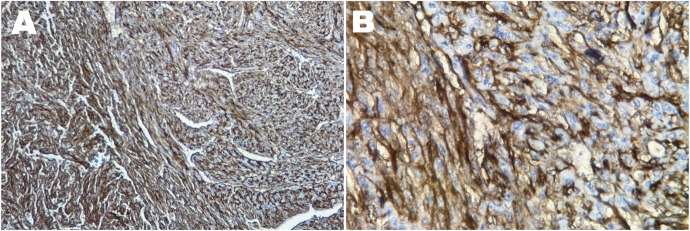
Immunohistochemical staining for CD31. A. Medium-power magnification (100×) demonstrating that almost all tumor cells are positive. B. High-power magnification (400×) showing strong membranous and cytoplasmic positivity in tumour cells, supporting endothelial differentiation.

**Fig. 5:**
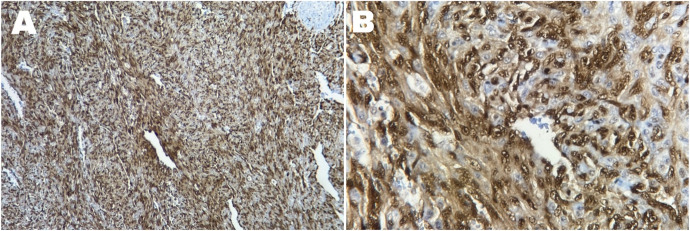
Immunohistochemical staining for ETS-related gene (ERG). A. Medium-power magnification (100×) demonstrating that almost all tumor cells are positive. B. High-power magnification (400×) demonstrating strong nuclear positivity in tumour cells, supporting endothelial differentiation.

**Fig. 6:**
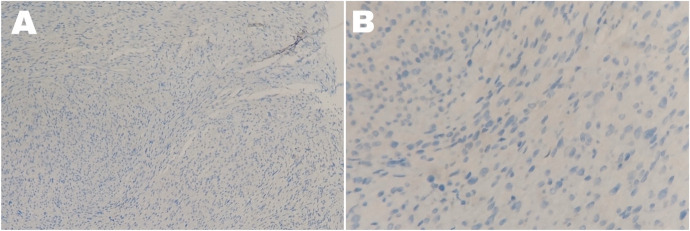
Immunohistochemical staining for inhibin. A. Medium-power magnification (100×) demonstrating that almost all tumor cells are negative. B. High-power magnification (400×) showing absence of tumour cell staining.

**Fig. 7:**
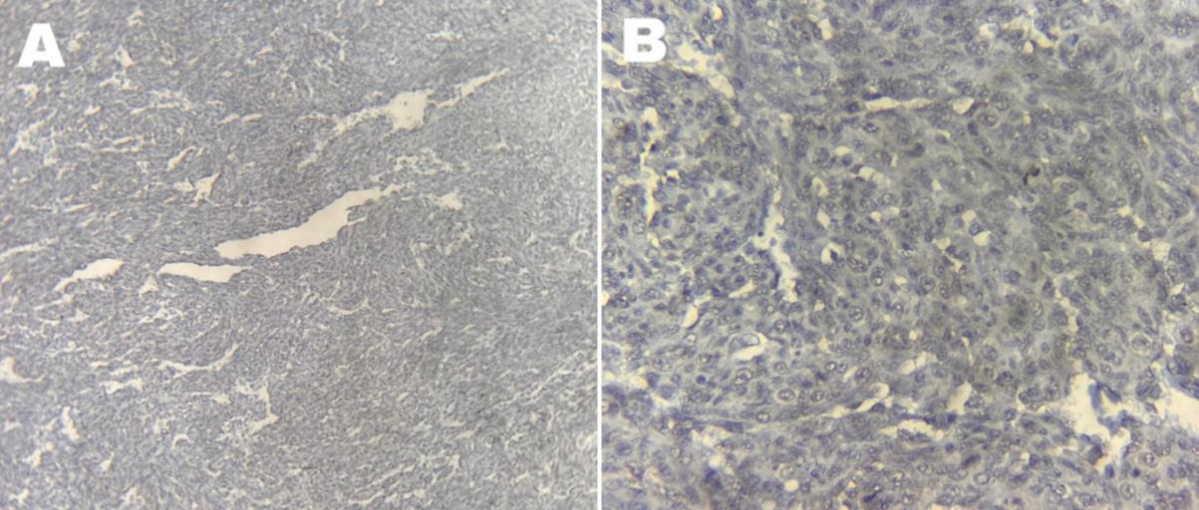
Immunohistochemical staining for BRCA2. A. Medium-power magnification (100×) demonstrating that almost all tumor cells are negative. B. High-power magnification (400×) demonstrating absence of nuclear staining in tumour cells.

The patient underwent total abdominal hysterectomy, bilateral salpingo-oophorectomy, omentectomy, and appendectomy. Gross pathology of the right ovary revealed a fragmented mass measuring up to 14.3 cm with tan-white, hemorrhagic, and necrotic areas (Fig 2). Microscopically, the tumour was composed of atypical spindle and epithelioid cells arranged in solid sheets with vasoformative architecture, including anastomosing vascular channels and extensive necrosis. The neoplastic cells exhibited marked pleomorphism, coarse chromatin, prominent nucleoli, and brisk mitotic activity (Fig 3). Vascular invasion was present, while lymphatic and perineural invasion were absent. The left ovary, uterus, and omentum were uninvolved. Histopathology was diagnostic of primary ovarian angiosarcoma, with adult granulosa cell tumour considered in the differential diagnosis. Tumour cells showed positivity for CD31 and ERG on additional IHC studies, confirming the diagnosis of ovarian angiosarcoma (Figs 4-6). Ki-67 demonstrated intense nuclear staining in approximately 50% of tumour cells. BRCA2 immunohistochemistry was negative (Fig 7). BRCA1 immunohistochemistry was not available at our hospital, and confirmatory molecular testing for BRCA1 and BRCA2 mutations by PCR could not be performed because of limited access to molecular diagnostic facilities in our resource-limited setting.

Following the discovery of the pelvic mass, a metastatic workup was initiated, beginning with a chest X-ray that revealed multiple well-defined, rounded nodules throughout both lungs, the largest measuring 0.7 cm, highly suggestive of pulmonary metastases. A contrast-enhanced abdominal and thoracic MSCT was then performed to assess the extent of the disease. Imaging demonstrated multiple hepatic and splenic metastases, mixed-type (nodular and pneumonic) pulmonary metastases, left renal atrophy with multiple simple cysts, and grade 2-3 hydronephrosis. No evidence of lymphadenopathy was observed. Subsequent head MRI also revealed an extracranial soft tissue mass in the right fronto-orbital region and multiple nodules throughout the left cerebral and bilateral cerebellar hemispheres, suggestive of brain metastases. Collectively, these findings indicate widespread metastatic disease involving the liver, spleen, lungs, and brain.

The patient was initially planned to undergo chemotherapy for ovarian angiosarcoma. However, treatment was deferred due to clinical deterioration and severe anemia (hemoglobin 6.7 g/dL), for which she received blood transfusions prior to reassessment. The following day, she developed melena and was scheduled for a colonoscopy. Given her overall decline and a transition in treatment goals to palliation, the colonoscopy was cancelled, and she was commenced on palliative chemotherapy with epirubicin and ifosfamide with mesna, which was administered for 2 cycles. Unfortunately, the patient died exactly 3 months after being diagnosed with ovarian angiosarcoma.

## Discussion

Angiosarcomas most often arise in the skin and soft tissues, while visceral involvement, including the female genital tract, is uncommon.3 OA is an exceptionally rare endothelial malignancy, accounting for fewer than 1% of ovarian cancers and 1%-2% of soft tissue sarcomas, with fewer than 60 cases reported worldwide.3,4,7 It typically affects premenopausal women, with a mean age of 31 years, although pediatric cases have been reported.7

Most OAs arise de novo, though they may occasionally be associated with other ovarian tumours such as teratomas, mucinous cystadenocarcinomas, or dermoid cysts.7 Metastatic involvement of the ovary from angiosarcoma of other sites is exceedingly uncommon, with fewer than 10 cases reported to date, primarily originating from cutaneous or breast primaries.4,8–11 These typically occur as part of hematogenous dissemination in advanced disease, often with widespread involvement of the viscera. In the present case, metastatic spread from the patient’s prior invasive ductal carcinoma (IDC) is unlikely due to the distinct histopathological and immunophenotypic profiles. Whereas IDC is an epithelial malignancy, angiosarcoma is a mesenchymal neoplasm of endothelial origin, characterized by anastomosing vascular channels lined by atypical endothelial cells that are immunopositive for CD31 and ERG. These findings strongly support a diagnosis of primary OA rather than a secondary metastatic lesion. Notably, this case also confirms the aggressive biological behaviour of OA, as evidenced by hepatic, splenic, pulmonary, and brain metastases at presentation, highlighting its tendency for early and widespread dissemination, with metastasis present in 67.9% of primary OAs. This is consistent with patterns observed in previously reported cases involving metastasis to the lungs, spleen, liver, bone marrow, and peritoneal cavity.12–16

Radiation exposure is a recognized risk factor for angiosarcoma, with post-radiation cases reported in the vulva, vagina, urinary bladder, rectum, mons pubis, omentum, pelvic bones, and, most frequently, the breast. Although the patient had undergone prior radiotherapy for breast cancer, it is highly unlikely that the ovarian angiosarcoma resulted from this radiation exposure. This is because the ovarian tumour developed outside the irradiated field of the left chest wall and had started being symptomatic while the patient was undergoing radiotherapy. Meanwhile, the latency period between radiotherapy and subsequent radiation-associated angiosarcomas is approximately 10 years, ranging from 1 to 26 years.17 Over the past 25 years, only one case of radiation-induced ovarian sarcoma has been reported, which was most consistent with fibrosarcoma, although this was not explicitly stated by the authors.18 No cases of radiation-induced ovarian angiosarcoma have been reported. Conversely, two patients who received pelvic irradiation for ovarian carcinoma and dysgerminoma later developed terminal ileal angiosarcoma, rather than ovarian involvement.19,20 This may be explained by oophorectomy performed as part of initial ovarian cancer management, which removes the target organ for subsequent radiation-induced malignancy. Interestingly, in a cohort of over 300,000 cancer patients, Virtanen et al. reported that all 19 cases of secondary angiosarcoma occurred exclusively in women, predominantly among those with breast or gynecologic primary malignancies, and that more than half of these secondary angiosarcomas had received prior radiotherapy. The study, however, did not specify the anatomical sites of these angiosarcomas. Notably, the study did not demonstrate a strong overall association between radiotherapy and angiosarcoma incidence, contrary to prior assumptions that radiation exposure is a predominant etiologic factor.21 These findings suggest that female hormonal factors or genetic predispositions, such as mutations commonly associated with breast and gynecological cancers, may contribute to angiosarcoma susceptibility in this subset of patients.

Multiple primary cancers (MPCs), first described by Warren and Gates in 1932, are defined as 2 or more distinct primary tumours arising in the same individual, either synchronously or metachronously, and not as metastases or recurrences.22,23 Approximately 62%-66% of MPCs are metachronous, with the breast representing the second most frequent initial primary site (19%) and the ovary being the second most common subsequent site (18%).23 The occurrence of multiple primary malignancies involving the breast and ovary often reflects an underlying hereditary predisposition, most notably germline mutations in the BRCA1 and BRCA2 genes. These mutations markedly increase lifetime risks for both breast and ovarian cancer.22 In a cohort of patients with metachronous breast and ovarian cancers, Tasca et al. found BRCA mutations in up to 68.5% of tested cases. However, their analysis was limited to epithelial ovarian carcinomas, as non-epithelial subtypes are rarely linked to hereditary breast-ovarian cancer syndromes.6

Although exceedingly uncommon, angiosarcomas have been reported in BRCA mutation carriers. West et al. described a BRCA2 mutation carrier who developed chest wall angiosarcoma after treatment for breast carcinoma.24 In addition, genomic analyses of radiation-associated breast angiosarcomas have identified alterations in genes involved in the BRCA-associated DNA repair pathway.25 However, the clinical significance of these findings remains uncertain, and their relevance to primary ovarian angiosarcoma has not been clearly established.

PARP inhibitors (PARPi) exploit defects in DNA repair pathways, particularly in tumours with BRCA1 or BRCA2 mutations. By blocking single-strand DNA break repair, PARP inhibitors induce the accumulation of DNA damage that cancer cells with impaired BRCA-mediated repair cannot repair, leading to the selective death of tumour cells. Although primarily used in BRCA-mutated ovarian, breast, and pancreatic cancers, several reports have described the use of PARP inhibitors in ovarian angiosarcoma.26 Ye et al. described 9 months of disease-free survival using olaparib, a PARP inhibitor, combined with a PD-L1 inhibitor, and Johnson and Argenta reported 20 months of recurrence-free survival with olaparib in a patient with somatic BRCA2 expression.7,27 Although these reports raise the possibility that defects in DNA repair pathways could have therapeutic implications in selected cases, this possibility remains hypothetical. In the present case, comprehensive molecular testing was not performed; hence, the relevance of PARP inhibition cannot be determined.

Although current evidence remains limited, reports have described angiosarcomas occurring in individuals with BRCA mutations. While BRCA2 testing was performed, the absence of BRCA1 testing and assessment of other homologous recombination repair genes represents an important limitation of this report, resulting in an incomplete molecular characterization of the tumor. Consequently, any potential association between the patient’s malignancies and underlying DNA repair abnormalities remains speculative. Additionally, as this is a single case report, causal relationships cannot be established, and the findings may not be generalizable. Further molecular characterization of ovarian angiosarcomas may improve understanding of their underlying biology and determine whether any relationship exists between these malignancies and hereditary cancer syndromes. Given the extreme rarity of these malignancies, larger studies through multi-institutional collaborations and centralized registries will be essential to achieve reliable conclusions.

## Data Availability

The data that support the findings of this study are available from the corresponding author upon reasonable request.
